# How has our primary-care NHS-IAPT provision for PTSD adapted to the pandemic? A service evaluation of recovery pre-COVID-19 and peri-COVID-19

**DOI:** 10.1186/s12875-024-02295-7

**Published:** 2024-02-12

**Authors:** Lilian Skilbeck, Daniela Antonie, Stephen Crane

**Affiliations:** East London NHS Foundation Trust, Newham Talking Therapies, Vicarage Lane Health Centre, 10 Vicarage Lane, E15 4ES Stratford, UK

**Keywords:** PTSD, COVID-19, Pandemic, Recovery, IAPT, NHS

## Abstract

**Background:**

Mental health issues have been an ongoing major cause of global disability exacerbated by the COVID-19 pandemic. The unique challenges have been the high contagiousness of COVID-19 and atypical PTSD presentations e.g., ICU-PTSD. This has led to increased demand on mental health services which have had to vary their provision for example working remotely vs. the traditional face-to-face. The pandemic has also exposed the preexisting health inequalities related to sociodemographic variables. In the UK, NHS-IAPT is the main primary-care provider which has suffered these repercussions. Research from COVID-19 and previous viral outbreaks has estimated an increase in the prevalence of PTSD. Although services have been urged to monitor their provision, research on PTSD remains scanty. The current NHS-IAPT service was concerned about these ramifications of the pandemic and also wished to address the gap in the research. The aim was to conduct an evaluation of the impact of the COVID-19 on PTSD recovery. The first question evaluated the impact, and the second question evaluated the associated variables.

**Methods:**

The study employed a quantitative data analysis method. Data were extracted and analysed from the electronic database, IAPTus. The study evaluated PTSD recovery rates during pre-pandemic and peri-pandemic periods. The comparisons determined the impact of the pandemic as well as what recovery variables were significant. The data were analysed statistically using both descriptive statistics and inferential statistics (t-test and Chi-square). The data were analyzed in reference to the national NHS-IAPT standards via NHS-Digital.

**Results:**

The findings suggest that the pandemic had no significant impact on overall PTSD recovery rates, which also aligned with the national standards. These recovery rates fell below the target national standard of 50% regardless of the pandemic. Several client, service and treatment variables were shown to be associated with PTSD recovery rates.

**Conclusions:**

This evaluation highlights a pre-existing problem around the persistently low PTSD recovery rates. It also identifies variables that warrant further research in order to improve PTSD service-provision and mitigate any long-term pandemic impacts. This study also provides information for other services wishing to enhance their PTSD recovery rates.

## Background

Mental health issues have been an ongoing major cause of global disability exacerbated by the Coronavirus disease 2019 (COVID-19) pandemic. There has been a correlated increase in demand for mental health services for post-traumatic stress disorder (PTSD) linked to the trauma impacts of COVID-19. Unique challenges of COVID-19 have been due to the high contagiousness of the virus which necessitated social isolation. Due to these repercussions, services have had to vary the way they offer therapy for example offering therapy remotely as opposed to the traditional face-to-face [1, 2]. The pandemic has also exposed the preexisting health inequalities related to sociodemographic variables. For example, COVID-19-related PTSD was more prevalent and exacerbated in older people, Black and Asian ethnic minorities and those with pre-existing mental health difficulties [3–5]. Other data have also suggested a higher prevalence in females and the unemployed [6–9]. For example, a longitudinal study by Sun et al. [10], showed the development of chronic PTSD symptoms after a year, which were exacerbated in females compared with males. Learning from COVID-19 and previous viral outbreaks suggests that PTSD is a major aftermath psychopathology [11–13]. As evidenced with COVID-19, the main predictors of developing PTSD include serious physical injury, imminent threat to life and high death toll. For example, the experience of being in an intensive care unit (ICU), witnessing high death rates during an outbreak as well as being in isolation can be precursors to the development of PTSD [14, 15]. An added challenge arising from COVID-19, as with previous outbreaks has been the emergence of atypical PTSD cases presenting to the service e.g., ICU-PTSD. Research from COVID-19 and previous viral outbreaks also predicts that the PTSD repercussions of the pandemic could evolve over a number of years. Hong et al. [16] demonstrated a 44.1% prevalence rate of PTSD in a population who had recovered from SARS after a period of 4 years. Several other studies have reported similar findings amongst the general population and healthcare workers [17–19]. Unlike previous viruses, COVID-19 was shown to be more contagious with a high fatality rate and required months of social isolation and lockdown measures which were challenging for the general population and served as catalysts for PTSD [5, 12]. Although services have also been urged to monitor their provision, research on PTSD remains scanty.

In the UK, those seeking help for PTSD symptoms will often present to primary-care as the primary service. The NHS Improving Access to Psychological Therapies (NHS-IAPT) or NHS-Talking Therapies service is the main primary-care provider which has suffered the repercussions of the pandemic. This service offers evidence-based talking therapy as recommended by the National Institute for Health and Care Excellence (NICE) guidelines [20]. In line with the IAPT-manual [21], outcomes are routinely measured in terms of recovery which is a way of quantifying the benefits of therapy. This is defined as moving from caseness (above threshold) to non-caseness (below threshold) on specified measures.

The current NHS-IAPT service is a large provider located in the third most populous, diverse and deprived Boroughs of London which suffered the impact of the pandemic on PTSD service provision. For example, they moved to remote delivery of therapy, using telephone or video appointments. Although this presented an opportunity to transform teletherapy, it also presented challenges. For example there were issues around digital health inequity where those with no facilities for digital communication were disadvantaged including older adults, Black and ethnic minorities, the socially disadvantaged and those with pre-existing mental health difficulties [22–24]. Although this was circumvented through the introduction of in-service digital suites, it was still met with the challenges of COVID-19 isolation rules. Research suggests that teletherapy is more demanding than face-to-face therapy across dimensions such as establishing a therapeutic relationship, isolation and barriers to conducting technical trauma-focused therapy skills [24]. Therefore, these adaptations also presented challenges around standardization of trauma-focused cognitive behavioural therapy (CBT) and eye movement desensitization and reprocessing (EMDR) or using interpreters remotely, which raised concerns on how these variables might impact PTSD recovery. The current NHS-IAPT service was concerned about the impact of the pandemic on their PTSD provision, and also wished to address the gap in the research. The aim was to conduct an evaluation of the impact of the COVID-19 on PTSD recovery. The first question evaluated the impact of Covid-19 on recovery rates. The second question evaluated what variables were associated with recovery.

## Methods

### Study design

The study employed a quantitative methodology using a cross-sectional correlational design.

### Sample

**Fig. 1:**
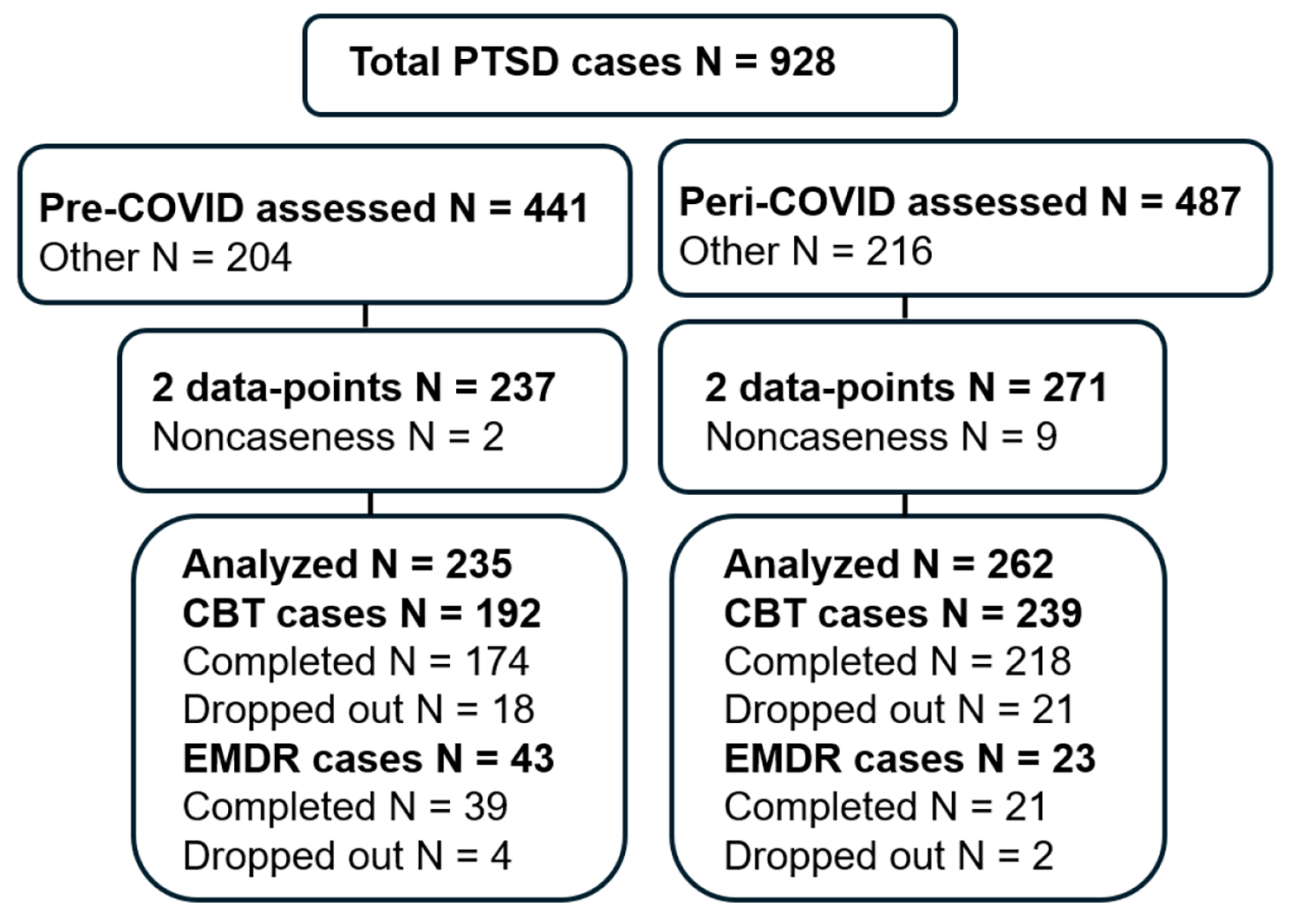
Sampling strategy showing the number of clients included in the study. Other indicates clients who were either referred on or did not take-up treatment. Completed and dropped out refer to the course of treatment

The sample consisted of data extracted from IAPTus [25], of clients who had accessed a London NHS-IAPT service for adults aged 18-years and above. As mentioned above, this service is situated in one of the most populated, diverse and deprived Boroughs of London. The sample was drawn from a population comprising 60% Black and Ethnic minority, 31% White and 9% other ethnic groups [26]. Of the total population, 65% listed English as their first language. The IAPTus is a clinical system which has inbuilt features to enable services to manage client recovery journeys from intake to discharge. The system records client demographics, attendance and outcomes. It also has inbuilt features to monitor therapist and service variables and allows for data to be extracted for service measurements. Data were extracted for cases who had received high intensity CBT or EMDR for PTSD. This included all clients who completed least two treatment sessions (coded as ‘assessment and treatment’ and/or ‘treatment’) within the specified periods, with the first session scores being recorded as the baseline. Exclusion criteria included those: initially scoring below caseness, with only one session-score, still in treatment. Those meeting the criteria were included in the study. For the pre-COVID-19 period data were cumulatively extracted from April 2019-March 2020 (*N* = 441 PTSD cases). For the peri-COVID-19 period, they were cumulatively extracted from April 2020-March 2021 (*N* = 487 PTSD cases). The sampling strategy is summarized in the flowchart shown in Fig. 1.

### Data collection

Data were collected in two stages: Data for all clients who received therapy for PTSD; data for those who recovered, unrecovered or dropped out. The data were extracted manually from the IAPTus database and transferred onto an MS-Excel spreadsheet. In line with the IAPT-manual, recovery was calculated based on paired-data outcomes for both the depression and the relevant anxiety or MUS measure. Recovery for PTSD was calculated based on the combination of the Patient Health Questionnaire-9 (PHQ-9; [27]) and the Posttraumatic Checklist for the DSM-5 (PCL-5; [28]). The Generalised Anxiety Disorder Assessment (GAD-7; [29]) score applies where the PCL-5 score is missing. Since the service routinely uses the PCL-5 as mandated for PTSD, GAD-7 scores did not apply. Recovery was defined as a shift in scores to less than 10 for the PHQ- (reliable change index of ≥ 6) and less than 32 for the PCL-5 (reliable change index of ≥ 10). The study data were evaluated against the national standards from NHS Digital which publishes aggregated data for national IAPT services every year [30].

### Variables

Given the above mentioned pandemic-imposed changes to service delivery, the included variables were waiting times, baseline symptom severity, demographics (age, ethnic-background, gender). The secondary variables were language status (interpreter usage), referral source (self vs. professional), service delivery methods (face-to-face vs. remote, trauma-focused CBT vs. EMDR number of sessions, number of episodes and attendance (Table 1). This selection of variables was also informed by previous NHS-IAPT research on variables related to recovery in general. For example, a report by Gyani et al. [31] suggested that baseline symptom severity was a key variable. These authors proposed that clients with higher baseline scores were less likely to recover and required more treatment sessions than those with lower ones. They also suggested that more treatment sessions were generally associated with recovery as were more experienced therapists. They, however, found no difference in recovery rates between self-referring compared with professionally referred clients. This report has been used as a guide for several other authors. For example, Clark et al. [32] suggested that number of sessions, wait times and social deprivation were related to recovery. Stochl et al. [33] identified gender, age, baseline symptom severity and social deprivation as related variables. Similar findings have also been reported by Vaillancourt et al. [34], who identified wait times, number of sessions and baseline symptom severity as related variables. Sauders et al. [35] also identified attendance-rates. Primary-care IAPT routinely publishes aggregated yearly outcomes. However, no in-depth evaluation at the service-level has been conducted in relation to PTSD recovery in the context of the pandemic, which is the concern of the current study.

**Table 1:** Variables included in the study

Variable	Description	Value
Recovery	PHQ-9 and PCL-5 below clinical cut-off	Recovered vs. unrecovered
Referral source	Source of referral	Self vs. professional referred
Wait to treatment	Difference between date of triage to date of first treatment session	Number of days
Number of episodes	Number of new episodes	Number of treatment starts with at least 2 sessions
Baseline symptom severity	Scores on symptom measures at first session	Score on PHQ-9 (range 10–27) and PCL-5 (range 32–80)
Number of sessions	Number of attended clinical contacts	Number of outcomed sessions
Treatment modality	Treatment intervention delivered	Trauma-focused CBT vs. EMDR
Attendance rate	Attendance to offered treatment sessions	Number of sessions cancelled or non-attended appointments (cancelled by client, cancelled by service, did not attend)
Interpreters	English or facilitated session in another language	Interpreter vs. non-interpreter session
Demographics	Sociodemographic client descriptors	Gender (male, female); Age (16–24, 25–39, 40–64, 65+); Ethnicity (white British, African, Bangladeshi, Any other white)

### Quantitative methods

Data were analysed statistically using both descriptive statistics and inferential statistics. The chosen statistical tests were the independent t-test for numerical means of service-user outcomes and Chi-square (χ2) for categorical data. The *P*-value for both was set at a significance level of ≤ 0.05. Analysis-1 compared recovery rates between pre-COVID-19 and peri-COVID-19 periods. Results were considered marginally significant for 0.05 < *P* ≤ 0.1. Analysis-2 evaluated the association between the selected variables and recovery.

### Ethical considerations

Ethical approval was sought from the East London Foundation Trust board of Ethics Committee (GECSE, G2109b). Service-user consent to the study and publication of anonymized data was obtained. The data management was conducted in line with the ethics guidelines.

## Results

### Analysis 1. Comparison of PTSD recovery rates between pre-COVID-19 and peri-COVID-19 periods?

#### Descriptive statistics

The number of cases at caseness who completed treatment for PTSD pre-COVID-19 was 235 and peri-COVID-19 was 262. Of these cases 98 and 100 cases recovered, respectively. The percent-recovery pre-COVID-19 was 98/235 × 100 = 41.7%. The percent-recovery peri-COVID-19 was 100/262 × 100 = 38.2%. These recovery rates fell below the target national standard of 50%. They were also comparable to the mean national averages of 38% and 40.3%, respectively [30]. The number of recovered cases for pre-COVID and Peri-COVID periods is summarized in Table 2.

**Table 2:** Number of cases recovered during pre-COVID vs. peri-COVID periods

	Recovery pre-COVID		Recovery peri-COVID	
	N	% of total	N	% of total
**Recovered**	98	41.7	100	38.2
**Unrecovered**	137	58.3	162	61.8
**Total**	235	100	262	100
		**% recovered**		**% recovered**
**Recovery by referral source**				
Self Professional	159 75	47.2 33.3	193 68	38.8 36.7
**Recovery by gender**				
Male Female	92 142	35.8 45.7	80 181	31.2 41.4
**Recovery by age category**				
16–24 25–39 40–64 65 +	61 101 65 7	32.7 40.5 49.2 71.0	87 116 56 8	36.8 38.7 41.7 0.0
**Recovery by ethnic-background**				
White British African Any other white Bangladesh	38 34 28 27	44.7 50 34.2 55.5	43 37 38 33	28.0 48.6 22.2 39.3
**Recovery by treatment modality**				
Trauma-focused CBT EMDR	192 43	38.9 36.6	239 23	37.1 34.0
**Recovery by language variables**				
With interpreter No interpreter	25 211	28.0 43.1	32 226	22.5 40.9

A χ2 analysis revealed that there was no significant difference in recovery between pre-COVID-19 and peri-COVID-19 periods (*P* = 0.422). The analysis also showed that service-users had significantly shorter wait times before their first session after triage peri-COVID-19 compared to pre-COVID-19 (*P* = 0.000012). There were also higher referrals for 16–24-year-olds (*P* = 0.020) and ‘any other white’ background (*P* = 0.040). There was also a higher use of interpreters peri-COVID-19 (0.0019). There were fewer referrals peri-COVID-19 for 65 + year-olds (*P* = 0.020). The data also revealed higher attendance rates peri-COVID-19 (*P* = 0.00133). There was also a marginally significant increase in self-referrals compared to professional referrals peri-COVID-19 (*P* = 0.077).

There was no significant difference in the other test variables including face-to-face vs. remote sessions, number of episodes, number of treatment sessions, baseline symptom-severity and gender between pre-COVID and peri-COVID periods.

### Analysis 2. Variables associated with PTSD recovery rates

#### Descriptive statistics

For both periods, a total of 497 PTSD cases completed therapy. Of these 198 (39.8) recovered and 299 (60.2) did not recover. The variables associated with overall recovery were analyzed.

The means and standard deviations of the measured variables are summarized in Table 3.

**Table 3:** Means and standard deviations of recovery variables. As recommended by the IAPT-manual, baseline symptoms are presented as a combination between the PHQ-9 and the PCL-5. *N* = 98 recovered and 137 unrecovered clients pre-COVID-19; total = 235. *N* = 100 recovered and 162 unrecovered clients peri-COVID-19; total = 262

Variable	Pre-COVID-19		Peri-COVID-19	
	Mean	SD	Mean	SD
**Wait to treatment (days)**				
Recovered Unrecovered	101 96	21.08 28.02	73 63	17.77 21.70
**Number of episodes**				
Recovered Unrecovered	1.97 2.24	0.51 0.70	1.88 2.39	0.30 0.62
**Number of treatment sessions**				
Recovered Unrecovered	11.64 9.10	1.46 2.01	12.52 8.96	1.98 2.43
**Non-attendance (sessions)**				
Recovered Unrecovered	61.0 108.6	13.01 47.60	79.3 166.0	19.73 76.39
**Baseline Symptom scores**				
Recovered				
PHQ-9 PCL-5	16.16 54.24	1.85 6.26	15.19 55.76	1.29 8.81
Unrecovered				
PHQ-9 PCL-5	19.20 60.22	1.28 4.04	17.62 62.28	1.42 5.35

A t-test analysis suggested a significant difference between recovered and unrecovered cases (*P* < 0.05) in relation to number of episodes, number of sessions and baseline symptom-severity. Recovered cases had fewer episodes than unrecovered cases (*P* = 0.047). They also received a larger number of sessions than unrecovered cases (*P* = 0.005). Recovered cases showed significantly lower initial baseline symptom-severity as indicated by their lower scores at the start of treatment on the PHQ-9 (*P* = 0.00018) and PCL-5 (0.010).

A χ2 analysis suggested a significant difference between recovered and unrecovered cases (*P* < 0.05) and referral source. There were also significantly higher recovery rates in those who self-referred compared to those who were referred by a professional (*P* = 0.0085).

A χ2 analysis suggested a marginally significant difference between recovered and unrecovered cases in relation to age, ethnicity and use of interpreters (*P* < 0.1). The 16–24 years age group showed lower recovery compared to the other age categories (*P* = 0.07). Similarly, the ‘any other white’ ethnic category showed lower recovery rates than the other ethnicity groups (*P* = 0.07). Those requiring interpreters also showed lower recovery rates than those who did not use an interpreter (*P* = 0.09). These marginal differences need to be interpreted with caution as they are prone to type-I errors.

There was no statistically significant difference between recovered and unrecovered cases in relation to face-to-face vs. remote sessions, wait times, attendance rates, CBT vs. EMDR and gender. Due to difficulties establishing therapist level of experience from the IAPTus, this variable was not analyzed. Similarly, group therapy for single incident PTSD could not be evaluated against individual therapy which also included multiple incident traumas which could not be distinguished from the available data.

## Discussion

The current findings suggest that the pandemic had a minimal impact on overall PTSD recovery rates. It, however, showed variations in referral rates which were related to age and ethnic-background. There was also an increase in the number of sessions requiring interpreters. The data suggest that the service was able to implement changes which were able to mitigate the impact on recovery between the pre-COVID and peri-COVID periods. For example, despite the increased demand on the service imposed by the pandemic, it was able to adapt by increasing its service provision using other modes of delivery and use of interpreters. The increase in the ‘other white’ ethnic category may have also correlated to the increase in the use of interpreters. What was also noteworthy was that there was an increase in referrals for the 18–24 year olds whilst the referral rates for the 65 + year olds decreased. The factors associated with these variables warrant further investigation. It is possible that the older adults may have been digitally excluded due to the increased use of remote sessions due to the pandemic. It also highlights the need for the service to identify other COVID-19 and non-COVID-19 related needs of this population as outlined in the IAPT Positive Practice Guide Older People [26]. Regardless of the pandemic, recovery generally fell below the target national standard of 50% both Pre-COVID (41.7%) and peri-COVID (38.2%). These recovery rates were comparable to the national averages of 38% and 40.3%, respectively.

In answer to the service concerns, this evaluation identified several variables associated with PTSD recovery rates. A noteworthy finding was the increase in the number of ‘any other white’ ethnic referrals, an increase in the number of sessions requiring the use of interpreters and an association between poorer recovery rates and interpreter-facilitated sessions compared to non-interpreter sessions. As outlined by Woodward et al. [36], use of interpreters can be challenging. Therefore, the service needs to further explore the causal-factors associated with these variables. This is particularly important to mitigate health inequalities given that the sample was drawn from a population where only 65% identified English as their first language. Upon reviewing the literature, no other study has been conducted to show language status as a key variable in PTSD recovery. Given the diverse societies which IAPT serves, this finding has implications for the current service as well as other NHS-IAPT services. Self-referral was also identified as a variable positively associated with recovery. It is possible that self-referring service-users are more motivated for change. It highlights the need for the service to further attend to service-users who may be referred by professionals as well as those with repeated episodes to identify the causal-factors. This is in line with Sauders et al. [35] also suggested managing nonattendance promotes recovery. Baseline symptom-severity and number of sessions were also shown to impact recovery. This is in agreement with the findings by Vaillancourt et al. [34] in relation to general recovery rates. Both research groups suggested that those with higher baseline scores would require more sessions. Although this seems like a helpful avenue, the current service would need to weigh up the demands and service resources. The service also needs to further explore the specific causal factors of the high baseline scores. The study also highlighted other relevant variables such as unemployment, therapist experience and the use of group therapy which were not evaluated in this study. Given that research suggests the significance of these variables, they also warrant a research evaluation.

### Strengths and limitations

A key strength of this study is that data were obtained directly from a client database, which added to accuracy in reflecting realistic outcomes. There was a clearly defined measure of recovery which measured the shift from caseness to noncaseness. This allowed for analyses which could be compared with national data from NHS Digital. A limitation is that the data were extracted from a large database meaning there was a chance for statistical error. Another limitation was that some findings were extracted from small numbers which limits generalisability and means that they should be interpreted with caution. As this was a correlational service evaluation, it provides information on the variables associated with recovery. However, it does not offer a case-effect explanation which limits the possibilities for problem solving. Regardless of this, the current study identified significant variables for further controlled research.

## Conclusions

The current study suggests that the service adapted to the pandemic with no significant impact on recovery rates. It highlights the persistently low PTSD recovery rates which fall below the national set target of 50%. It also identifies variables associated with PTSD recovery rates, which provides significant information for the service to further elucidate the causal-effects. This study could also inform other services wishing to enhance their PTSD recovery rates.

## Data Availability

The data underlying this article will be shared on reasonable request to the corresponding author.

## Abbreviations

COVID-19: Coronavirus Disease 2019
CQC: Care Quality Commission
IAPT: Improving Access to Psychological Therapies
NHS: National Health Service
NICE: National Institute for Health and Care Excellence
PTSD: Posttraumatic stress disorder
